# Inhibition of late sodium current suppresses calcium-related ventricular arrhythmias by reducing the phosphorylation of CaMK-II and sodium channel expressions

**DOI:** 10.1038/s41598-017-01056-0

**Published:** 2017-04-20

**Authors:** Xiao-Hong Wei, Shan-Dong Yu, Lu Ren, Si-Hui Huang, Qiao-Mei Yang, Ping Wang, Yan-Peng Chu, Wei Yang, Yan-Sheng Ding, Yong Huo, Lin Wu

**Affiliations:** 1 Department of Cardiology, Peking University First Hospital, Beijing, 100034 China; 2 Key Laboratory of Molecular Cardiovascular Sciences, Ministry of Education, Beijing, 100191 China

## Abstract

Cardiac arrhythmias associated with intracellular calcium inhomeostasis are refractory to antiarrhythmic therapy. We hypothesized that late sodium current (*I*_Na_) contributed to the calcium-related arrhythmias. Monophasic action potential duration at 90% completion of repolarization (MAPD_90_) was significantly increased and ventricular arrhythmias were observed in hearts with increased intracellular calcium concentration ([Ca^2+^]_i_) by using Bay K 8644, and the increase became greater in hearts treated with a combination of ATX-II and Bay K 8644 compared to Bay K 8644 alone. The prolongations caused by Bay K 8644 and frequent episodes of ventricular tachycardias, both in absence and presence of ATX-II, were significantly attenuated or abolished by late *I*_Na_ inhibitors TTX and eleclazine. In rabbit ventricular myocytes, Bay K 8644 increased *I*_CaL_ density, calcium transient and myocyte contraction. TTX and eleclazine decreased the amplitude of late *I*_Na_, the reverse use dependence of MAPD_90_ at slower heart rate, and attenuated the increase of intracellular calcium transient and myocyte contraction. TTX diminished the phosphorylation of CaMKII-δ and Na_v_ 1.5 in hearts treated with Bay K 8644 and ATX-II. In conclusion, late *I*_Na_ contributes to ventricular arrhythmias and its inhibition is plausible to treat arrhythmias in hearts with increased [Ca^2+^]_i_

## Introduction

An increase in intracellular calcium concentration ([Ca^2+^]_i_), i.e. calcium overload, is commonly seen in many pathological and pharmacological conditions, including tachycardia, acquired heart diseases (heart failure, ischemia/reperfusion, cardiomyopathy)^1–3^, inherited calcium channelopathies (long QT syndrome 8 and catecholaminergic polymorphic ventricular tachycardia, CPVT)^4, 5^, and post-administration of digitalis and catecholaminergic agents, etc^6, 7^. Increased [Ca^2+^]_i_-related diseases are characterized by distinctive cardiac electrical and mechanistic manifestations, including life-threatening ventricular arrhythmias which are difficult to evaluate and lack effective treatments^8, 9^. Recent clinical reports indicate that the late sodium current (late *I*_Na_) inhibitors ranolazine and mexiletine are effective, at least in part, in preventing or treating increased [Ca^2+^]_i_-associated cardiac arrhythmias, including LQTs 8 and myocardial hypertrophy^10, 11^, etc. The mechanisms underlying these treatments remain to be investigated.

Our previous study indicated that endogenous late *I*_Na_ contributed to ventricular arrhythmias associated with bradycardia and administration of QT prolonging drugs that inhibits rapidly activated delayed rectifier K^+^ current (*I*_Kr_)^12–14^. Late *I*_Na_ was increased in cardiomyocytes when [Ca^2+^]_i_ was high via the subsequent activation of Ca^2+^/calmodulin dependent protein kinase (CaMK)II and protein kinase C (PKC) pathways, and the resulting increase in intracellular sodium concentration would further elevate [Ca^2+^]_i_ via reversed sodium-calcium exchange^15^. This positive vicious cycle may enhance the proarrhythmic characteristics of increased [Ca^2+^]_i_ in the heart. (S)-(-)-Bay K 8644 (Bay K 8644), a L-type Ca^2+^ channel agonist, accelerated Ca^2+^ current activation/inactivation kinetics and Ca^2+^ peak current amplitude by twofold^16^. In addition, sea anemone toxin (ATX)-II at concentrations of 1–3 nM increased late *I*_Na_, and while it did not cause any ventricular arrhythmias itself, it did potentiate the risk of proarrhythmic drugs in rabbit isolated hearts^17^.

In this study, we hypothesized that endogenous and enhanced late *I*_Na_ might contribute to the intracellular Ca^2+^ overload-associated cardiac arrhythmias and myocardial dysfunction in a synergistic mode in association with upregulation of phosphorylation of CaMK-II and sodium channel. This effect is enhanced by the self-promoting effects of the previously described positive feedback cycle for late *I*_Na_. Increases in [Ca^2+^]_i_ and late *I*_Na_ were artificially achieved by infusions of Bay K 8644 and ATX-II, respectively^18^. The results of this study may prove helpful in explaining the mechanisms by which sodium channel inhibitors (mexiletine and flecainide) are effective in treating patients with LQTs 8 and CPVT. We speculate that selective late *I*_Na_ inhibitors will be useful for preventing or treating cardiac arrhythmias associated with increased [Ca^2+^]_i_, as well as for cardiac dysfunction in clinical settings. Tetrodotoxin (TTX), at a concentration of 1 µM, has been reported to inhibit late *I*_Na_ with minimal inhibition on peak *I*_Na_^19^. Eleclazine (GS-6615), a novel selective late *I*_Na_ inhibitor, has been shown to be potentially effective in treating arrhythmias in patients with LQTs 3 and other kinds of arrhythmias^20^. Therefore, TTX and eleclazine were used in this study to determine the contribution of late *I*_Na_.

## Results

### Prolongation of left ventricular monophasic action potential duration at 90% completion of repolarization (MAPD_90_) by Bay K 8644 was concentration-dependent, and was greater in hearts treated with ATX-II

In hearts paced at a cycle length (CL) of 1000 ms, left ventricular epicardial (epi-) and endocardial (endo-) MAPD_90_ were 173.8 ± 4.1 and 199.7 ± 5.1 ms, respectively. Bay K 8644 (10–300 nM, n = 11) increased both the epi- and endo-cardial MAPD_90_ in concentration-dependent manners by 42 ± 4 and 50 ± 5 ms, respectively.

**Figure 1:**
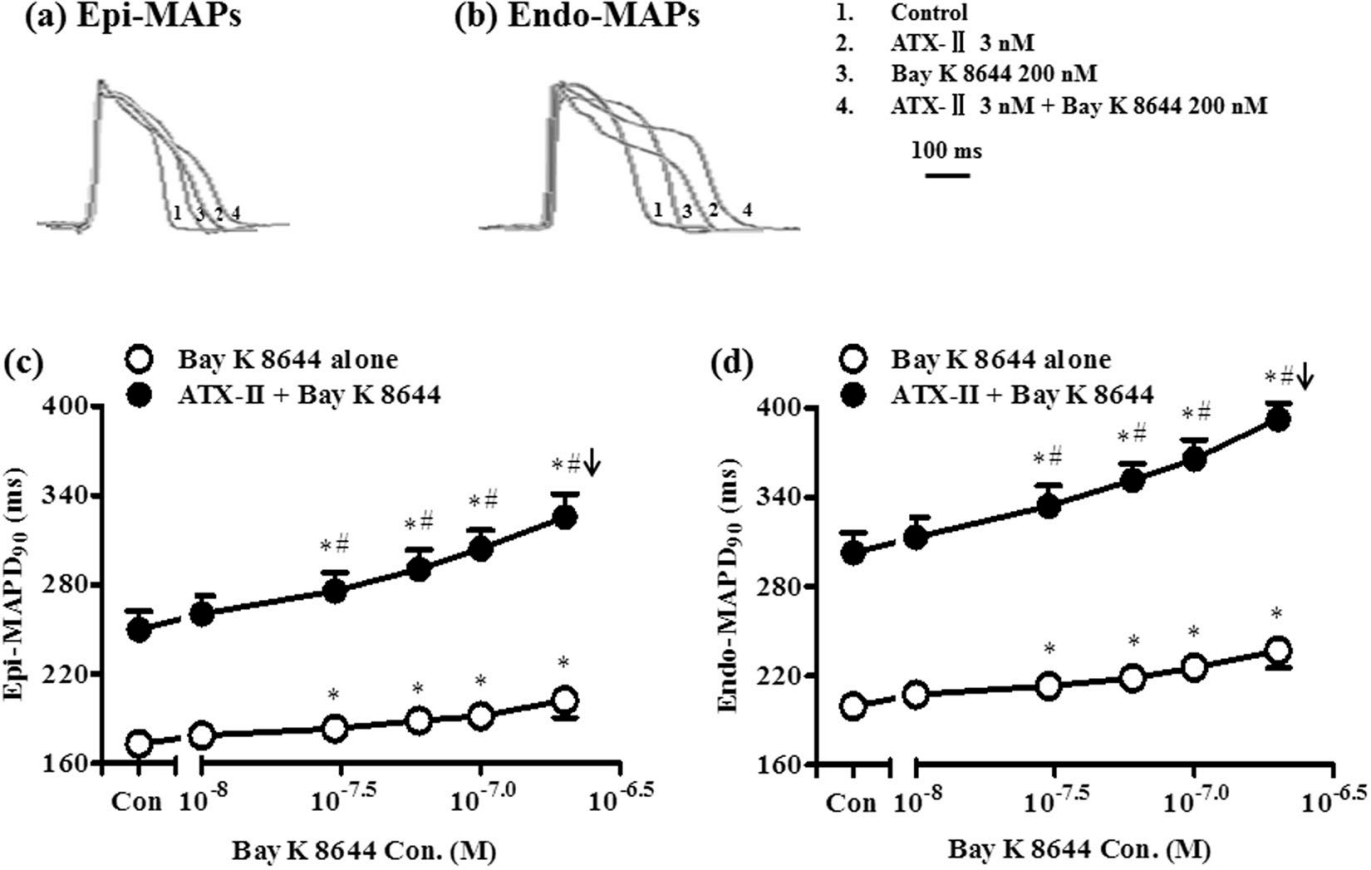
Prolongation of left ventricular monophasic action potential duration (MAPD_90_) by Bay K 8644 in the absence and presence of ATX-II. (**a**) and (**b**) Representative recordings of MAPs recorded from the epi- (**a**) and endo- (**b**) myocardium of the left ventricular wall in serially exposed to no drug (control), ATX-II (3 nM), Bay K 8644 (200 nM), and ATX-II (3 nM) plus Bay K 8644 (200 nM). (**c**) and (**d**) Concentration-dependent increases by Bay K 8644 of left ventricular epi- (**c**) and endo- (**d**) MAPD_90_ in absence (n = 11) and presence (n = 7) of ATX-II. ^*^ *P* < 0.05 compared with 0 nM Bay K 8644 either in absence or presence of ATX-II; ^#^ *P* < 0.05, the increase of MAPD_90_ by Bay K 8644 alone vs ATX-II plus Bay K 8644 at the same concentration. Arrows indicate ventricular tachycardia (VT) occurred at or above the concentration indicated.

In hearts with increased late *I*_Na_, i.e., after ATX-II administration, 3 nM ATX-II prolonged epi- and endo-MAPD_90_ by 68.5 ± 9.2 and 91.7 ± 11.8 ms, respectively (n = 7, Fig. 1(c) and (d)). In the continuous presence of 3 nM ATX-II, Bay K 8644 (10–200 nM) prolonged MAPD_90_ in concentration-dependent manners, and the increase of both epi- and endo- MAPD_90_ by Bay K 8644 (200 nM) were significantly greater in hearts treated with a combination of 3 nM ATX-II (above the value of ATX-II alone, equal to 70.4 ± 8.4 and 90.0 ± 9.4 ms, resp.), than that in hearts treated with Bay K 8644 alone (above the value of control, equal to 29.2 ± 3.3 and 37.3 ± 3.9 ms, resp. *P *<0.05) (Fig. 1(c) and (d)). Consequently, the slope of the concentration response curve for Bay K 8644 to increase MAPD_90_ was greater in the presence than in the absence of ATX-II (Fig. 1(c) and (d)). Representative AP traces in groups of control, ATX-II (3 nM), Bay K 8644 (200 nM), and ATX-II (3 nM) plus Bay K 8644 (200 nM) were shown in Fig. 1(a) and (b).

### Inhibition of late *I*_Na_ by TTX and eleclazine reduced the prolongation of MAPD_90_ by Bay K 8644 both in absence and presence of ATX-II

**Figure 2:**
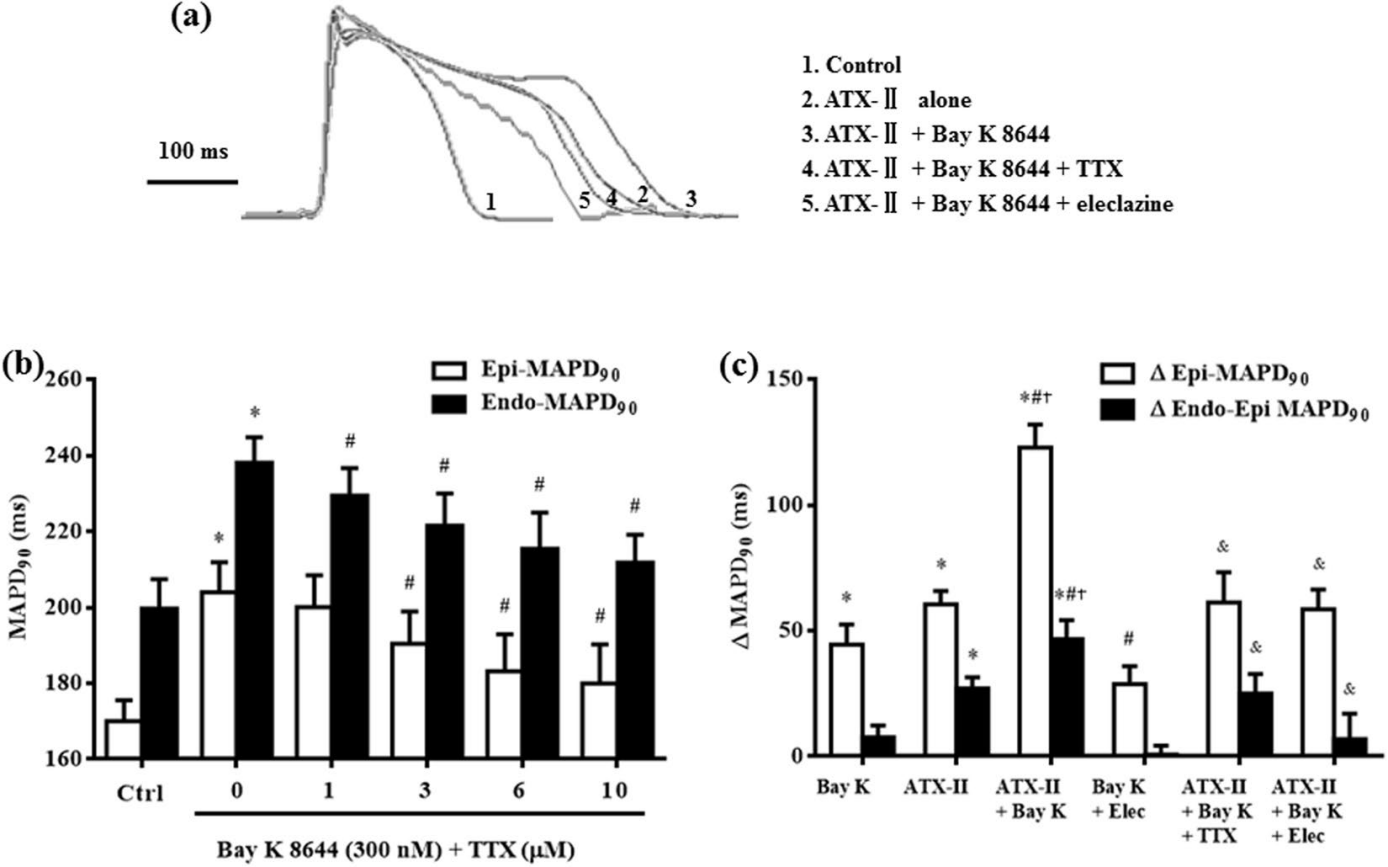
Reduction of MAPD_90_ prolongation by Tetrodotoxin (TTX) and eleclazine (Elec) in hearts treated with Bay K 8644 alone and ATX-II plus Bay K 8644. (**a**) Representative recordings of MAPs recorded from the epicardium of the left ventricular wall in serially exposed to no drug (control, curve 1), ATX-II (3 nM, curve 2), ATX-II plus Bay K 8644 (200 nM, curve 3), ATX-II plus Bay K 8644 plus TTX (1 µM, curve 4), and ATX-II plus Bay K 8644 plus eleclazine (10 µM, curve 5). (**b**) Concentration-dependent decreases by TTX of epi-MAPD_90_ and endo-MAPD_90_ in the presence of 300 nM Bay K 8644 (n = 7). (**c**) Effect of TTX (1 µM) and eleclazine (10 µM) on the increase of Δ epi-MAPD_90_ and Δ endo-epi MAPD_90_ induced by Bay K 8644 in absence and presence of ATX-II. ^*^ *P* < 0.05 vs. control (ctrl); ^#^ *P* < 0.05 vs. Bay K 8644; ^†^ *P* < 0.05 vs. ATX-II; ^&^ *P* < 0.05 vs. ATX-II plus Bay K 8644.

In hearts treated with Bay K 8644 alone (300 nM), TTX (at concentrations of 0.3, 0.6 and 1 µM) substantially reduced the increases of epi- and endo- MAPD_90_ (Fig. 2(b)). The reduction of epi- and endo- MAPD_90_ by 1 µM TTX were 24.1 ± 7.6 and 26.3 ± 1.9 ms, respectively (Fig. 2(b)). In hearts treated with both ATX-II (3 nM) and Bay K 8644 (200 nM), TTX (1 µM) caused significantly greater reduction in the prolongation of epi- and endo- MAPD_90_, with decreases of 61.7 ± 6.8 and 83.2 ± 10.1 ms, respectively (Fig. 2(c)). Similar to TTX, eleclazine also significantly decreased Bay K 8644-induced prolongation of epi- and endo- MAPD_90_ both in absence and presence of ATX-II (Fig. 2(c))_._ Compared with Bay K 8644 alone, the reduction of epi- and endo- MAPD_90_ by 10 µM eleclazine were 15.9 ± 2.6 and 23.7 ± 2.4 ms, resp. (Fig. 2(c), *P* < 0.05 vs. Bay K 8644 alone, n = 6). In the presence of ATX-II, the reduction of epi- and endo- MAPD_90_ by eleclazine were 64.3 ± 7.6 and 104.1 ± 3.6 ms, respectively (Fig. 2(c), *P* < 0.05 vs. ATX-II + Bay K 8644, n = 8). In addition, ATX-II plus Bay K 8644 significantly enhanced the transmural dispersion of MAPD_90_ (Δ Endo-Epi MAPD_90_), which was also reversed by TTX and eleclazine (Fig. 2(c)). Representative AP traces were shown in Fig. 2(a).

### Inhibition of late I_Na_ by TTX abolished Bay K 8644-induced ventricular arrhythmias both in absence and in presence of ATX-II

**Figure 3:**
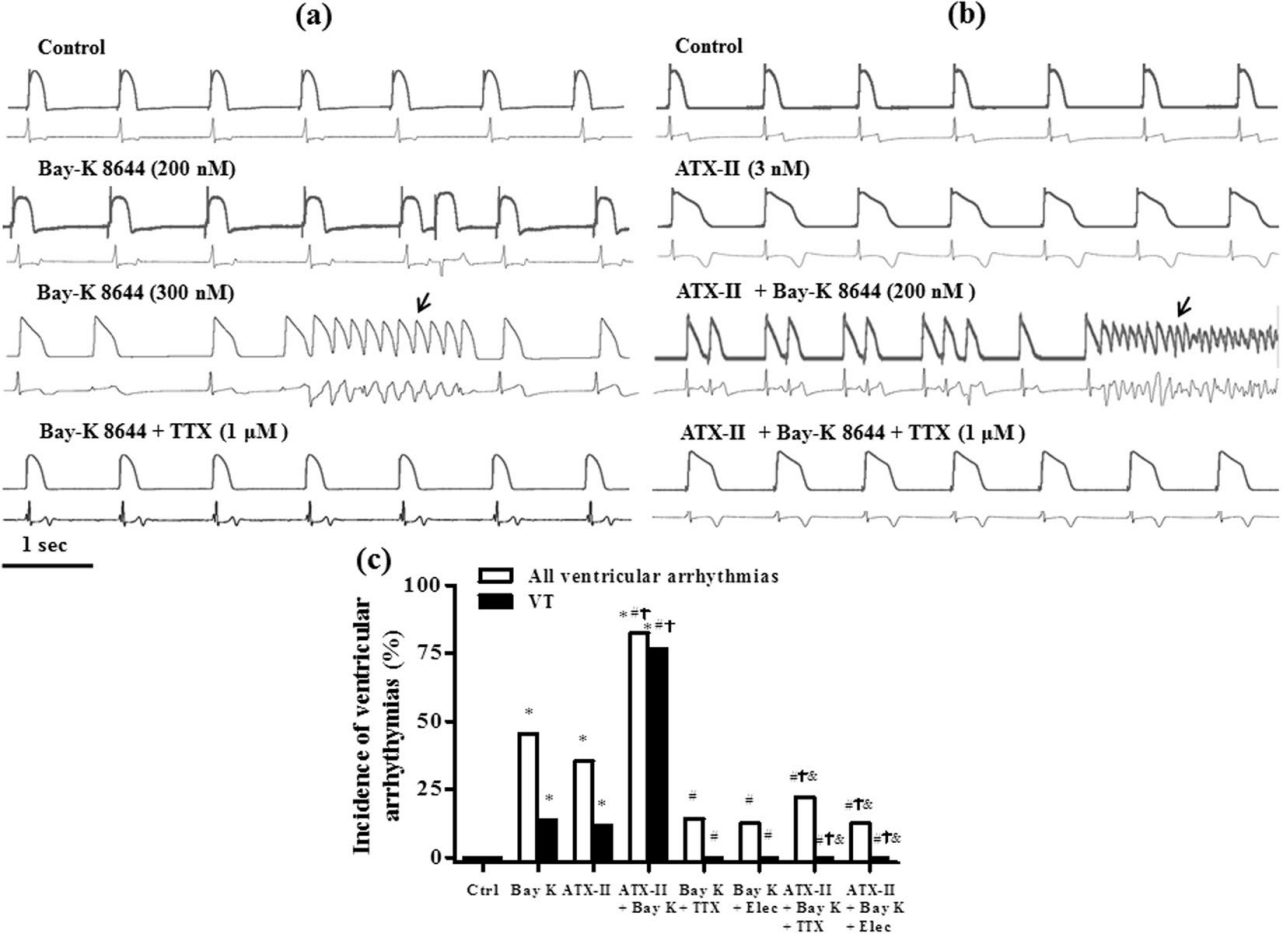
TTX and eleclazine (Elec) abolished ventricular arrhythmias induced by Bay K 8644 both in absence (**a**) and presence (**b**) of ATX-II. Representative recordings of MAPs (upper records in each panel) and ECG (lower records in each panel) were recorded simultaneously in control, Bay K 8644 (200 and 300 nM, **a**) or ATX-II-treated hearts (**b**) before and after treatment with TTX. Arrows indicate an episode of VT. The incidence of all ventricular arrhythmias and VT are presented in panel c. ^*^ *P* < 0.05 vs. ctrl; ^#^ *P* < 0.05 vs. Bay K 8644; ^†^ *P* < 0.05 vs. ATX-II; ^&^ *P* < 0.05 vs. ATX-II plus Bay K 8644.

Early after depolarization (EAD), extra-ventricular beats (EVBs), and ventricular tachycardia (VT) were not observed in any heart at control condition. Bay K 8644 alone caused EVBs in 10 out of 22 hearts (45.5%, Fig. 3(a) and (c)) and episodes of VT in 3 out of 22 hearts (13.6%, Fig. 3(c)) at a concentration of 200 nM, and frequent EVBs in 20 out of 22 hearts (90.9%) and episodes of VT in 6 out of 22 hearts (27.3%) at a concentration of 300 nM (Fig. 3(a) and (c)). In contrast, in the presence of 3 nM ATX-II, Bay K 8644 (200 nM) caused frequent EVBs in 14 out of 17 hearts (82.4%, Fig. 3(b) and (c)), and polymorphic VT in 13 out of 14 hearts (76.5%, Fig. 3(b) and (c)). These ventricular arrhythmias, including EVBs, and VT, but not ventricular fibrillation in 4 hearts, evoked by either Bay K 8644 alone or ATX-II + Bay K 8644, were attenuated and/or abolished by either 1 µM TTX (n = 14 for Bay K 8644 + TTX, and n = 9 for ATX-II + Bay K 8644 + TTX) or 10 µM eleclazine (n = 8 for Bay K 8644 + eleclazine, and n = 8 for ATX-II + Bay K 8644 + eleclazine) (Fig. 3(c)).

### TTX and eleclazine decreased the reverse use dependence (RUD) of Bay K 8644 on left ventricular MAPD_90_, and abolished Bay K 8644-induced ventricular arrhythmias at longer CL

**Figure 4:**
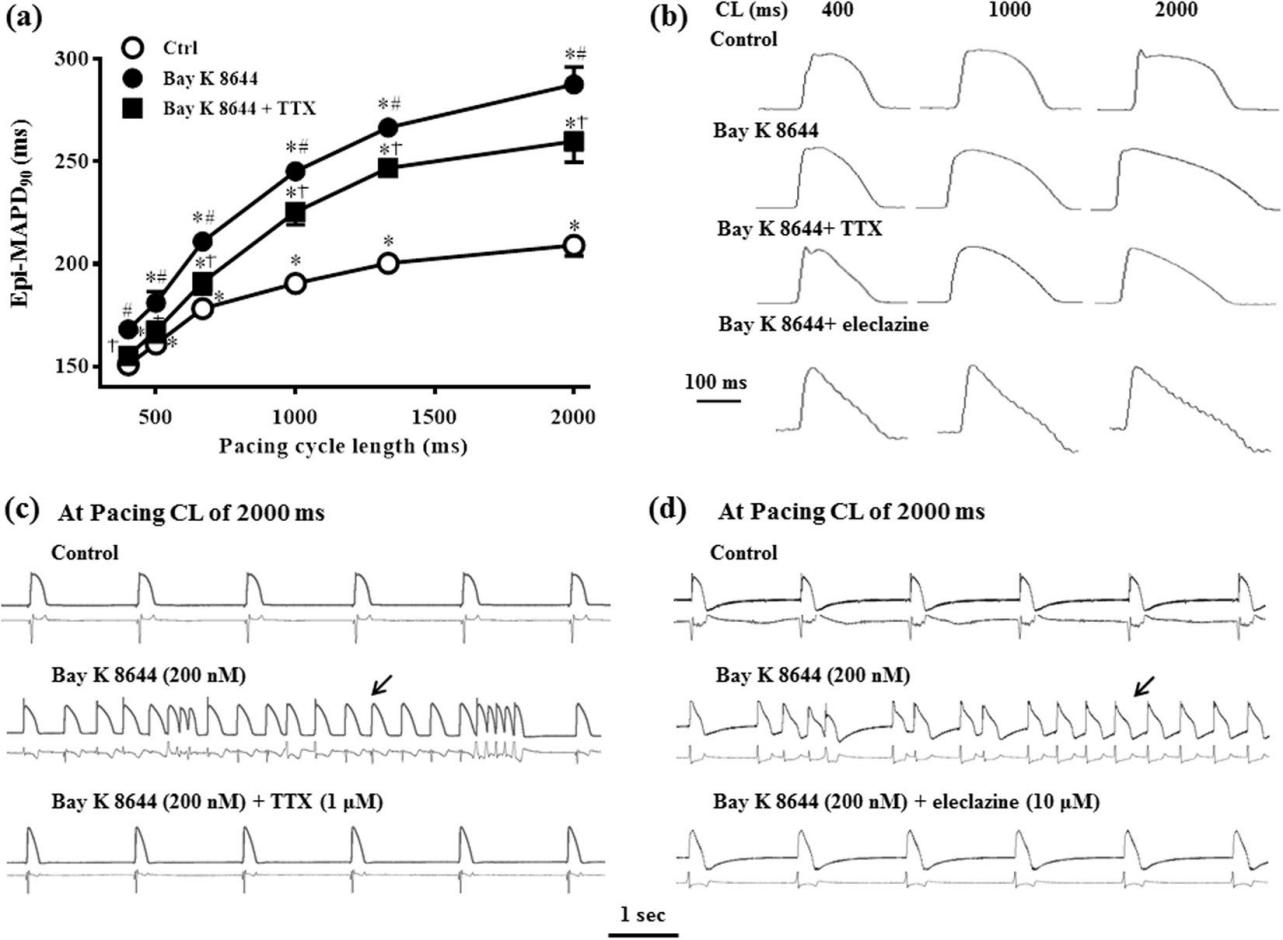
Effects of TTX and eleclazine on the reverse use dependence (RUD) of MAPD_90_ caused by Bay K 8644. (**a**) Values of MAPD_90_ were measured in the absence (Ctrl) and presence of Bay K 8644 (200 nM), Bay K 8644 (200 nM) plus TTX (n = 11). ^*^ *P* < 0.05 vs. cycle length (CL) of 400 ms; ^#^ *P* < 0.05 vs. Ctrl at the same CL; ^†^ *P* < 0.05 vs. Bay K 8644 alone at the same CL. (**b**) Representative AP traces obtained when hearts were paced at CLs of 400 ms, 1000 ms and 2000 ms in control, Bay K 8644 alone, Bay K 8644 plus TTX, and Bay K 8644 plus eleclazine. (**c**) and (**d**) Representative recordings of MAPs (upper records in each panel) and ECG (lower records in each panel) were recorded at pacing CL of 2000 ms in control, Bay K 8644 (200 nM), Bay K 8644 + TTX (1 µM), and Bay K 8644 + eleclazine (10 µM). Arrows indicate an episode of VT.

In the absence of drug (control), MAPD_90_ of the left ventricular epicardium (epi-) MAPD_90_ at CL of 400 ms was 150.8 ± 2.3 ms. As the pacing CL was prolonged to 500, 667, 1000, 1333 and 2000 ms, epi-MAPD_90_ was significantly increased in an RUD manner by 10.1 ± 1.0, 27.4 ± 1.3, 39.7 ± 2.3, 49.5 ± 2.3 and 58.4 ± 4.1 ms to 160.9 ± 3.2, 178.2 ± 3.3, 190.5 ± 4.1, 200.3 ± 3.7 and 209.2 ± 5.2 ms, respectively (n = 11, *P  *<0.05, Figs 4(a) and Supplementary Fig. S1).

In hearts treated with Bay K 8644 (200 nM), the prolongation of epi-MAPD_90_ were increased at all stimulation rates (n = 11, *P* < 0.05, Fig. 4(a)), and the increase was greater at longer CLs (e.g., 78.5 ± 5.5 ms at a CL of 2000 ms) than at shorter CLs (17.3 ± 2.3 ms at a CL of 400 ms, *P* < 0.05).

In the continued presence of Bay K 8644 in same group of hearts, late *I*_Na_ inhibitors TTX and eleclazine significantly attenuated the RUD of epi-MAPD_90_, especially at longer CLs (n = 11 and 5, *P* < 0.05, Fig. 4(a) and Supplementary Fig. S1, representative AP traces were shown in Fig. 4(b)).

EADs, EVBs, and VT were not observed in all hearts at shorter CLs of 400, 500, and 667 ms in absence and presence of Bay K 8644. At CLs of 1000, and 1333 ms, Bay K 8644 (200 nM) only caused EVBs and a few episodes of VT. In contrast, frequent EVBs and episodes of VT in 10 out of 13 hearts (76.9%) were induced by Bay K 8644 at a CL of 2000 ms. These ventricular arrhythmias, including EADs, EVBs, VTs evoked by Bay K 8644 at all pacing CLs were attenuated by TTX and eleclazine (Fig. 4(c) and (d)).

### Attenuation by TTX of the enhanced myocardial contraction and the increase of intracellular calcium transient induced by Bay K 8644 in single ventricular myocytes

**Figure 5:**
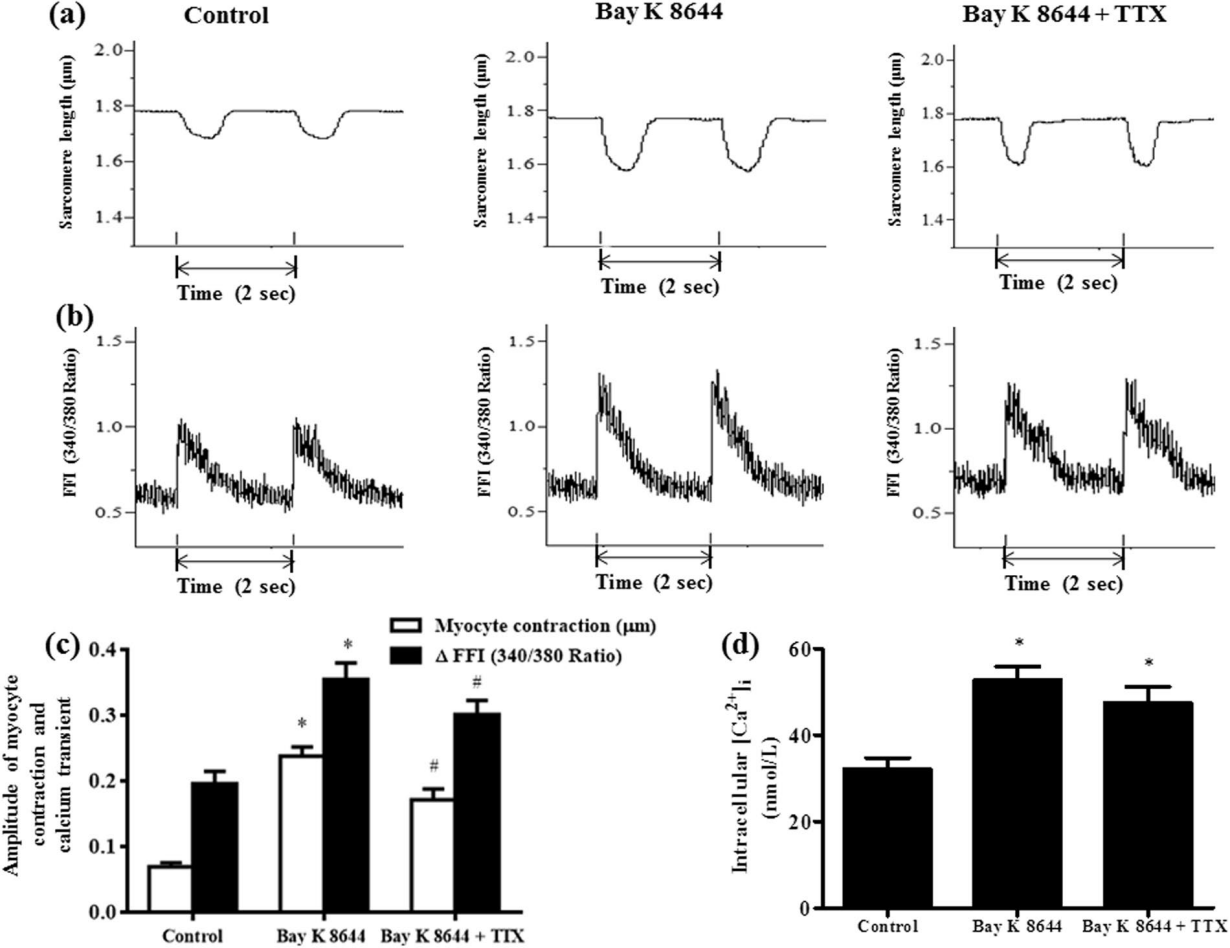
Effects of TTX (1 µM) on cardiomyocyte contraction function and intracellular calcium transient in the presence of Bay K 8644 (300 nM). (**a**) and (**b**) Representative recordings of myocardial sarcomere length (**a**) and intracellular calcium transients (**b**) under different conditions. (**c**) The myocardial contraction amplitude (n = 25), and calcium transient amplitude (n = 16) in control, Bay K 8644, and Bay K 8644 plus TTX. (**d**) The intracellular calcium concentration ([Ca^2+^]_i_) in myocardial cells in control, Bay K 8644, and Bay K 8644 plus TTX, n = 16. ^*^ *P* < 0.05 vs. control; ^#^ *P* < 0.05 vs. Bay K 8644.

The average sarcomere lengths at diastolic and systolic phase in single ventricular myocytes in control condition were 1.85 ± 0.01 and 1.78 ± 0.01 µm, respectively. Bay K 8644 (300 nM) significantly shortened the diastolic and systolic sarcomere lengths to 1.83 ± 0.01 and 1.59 ± 0.02 µm, respectively (n = 25, *P* < 0.05). Additionally, myocyte contraction amplitude was enhanced when treated with 300 nM Bay K 8644, from 0.07 ± 0.01 to 0.24 ± 0.01 μm (Fig. 5(a) and (c)). TTX at 1 µM depressed the increases by Bay K 8644 on myocyte contraction amplitude by 27.8%, to 0.17 ± 0.02 µm (Fig. 5(c), representative sarcomere lengths were shown in Fig. 5(a)).

Diastolic and systolic calcium fura-2 AM fluorescence intensities (FFI, 340/380 Ratio), and the intracellular [Ca^2+^]_i_ were increased after administration of 300 nM Bay K 8644 (Fig. 5(c) and (d), n = 16, *P* < 0.05). At this concentration, Bay K 8644 significantly increased the FFI difference between diastolic and systolic phases (ΔFFI) (Fig. 5(b) and (c)). Bay K 8644 increased the intracellular calcium transient amplitude and intracellular [Ca^2+^]_i_ from 0.20 ± 0.02 (ΔFFI, 340/380 ratio) and 32.17 ± 2.61 nM to 0.36 ± 0.03 (ΔFFI, 340/380 ratio) and 52.76 ± 3.23 nM, respectively (n = 16, *P* < 0.05). In the continued presence of Bay K 8644, 1 µM TTX inhibited the increases in calcium transient amplitude by 15.0% (Fig. 5(c), representative intracellular calcium transient in each group was shown in Fig. 5(b)).

### Bay K 8644 increased *I*_CaL_ but not late *I*_Na_, and endogenous and ATX-II-augmented late *I*_Na_ were attenuated by both TTX and eleclazine

**Figure 6:**
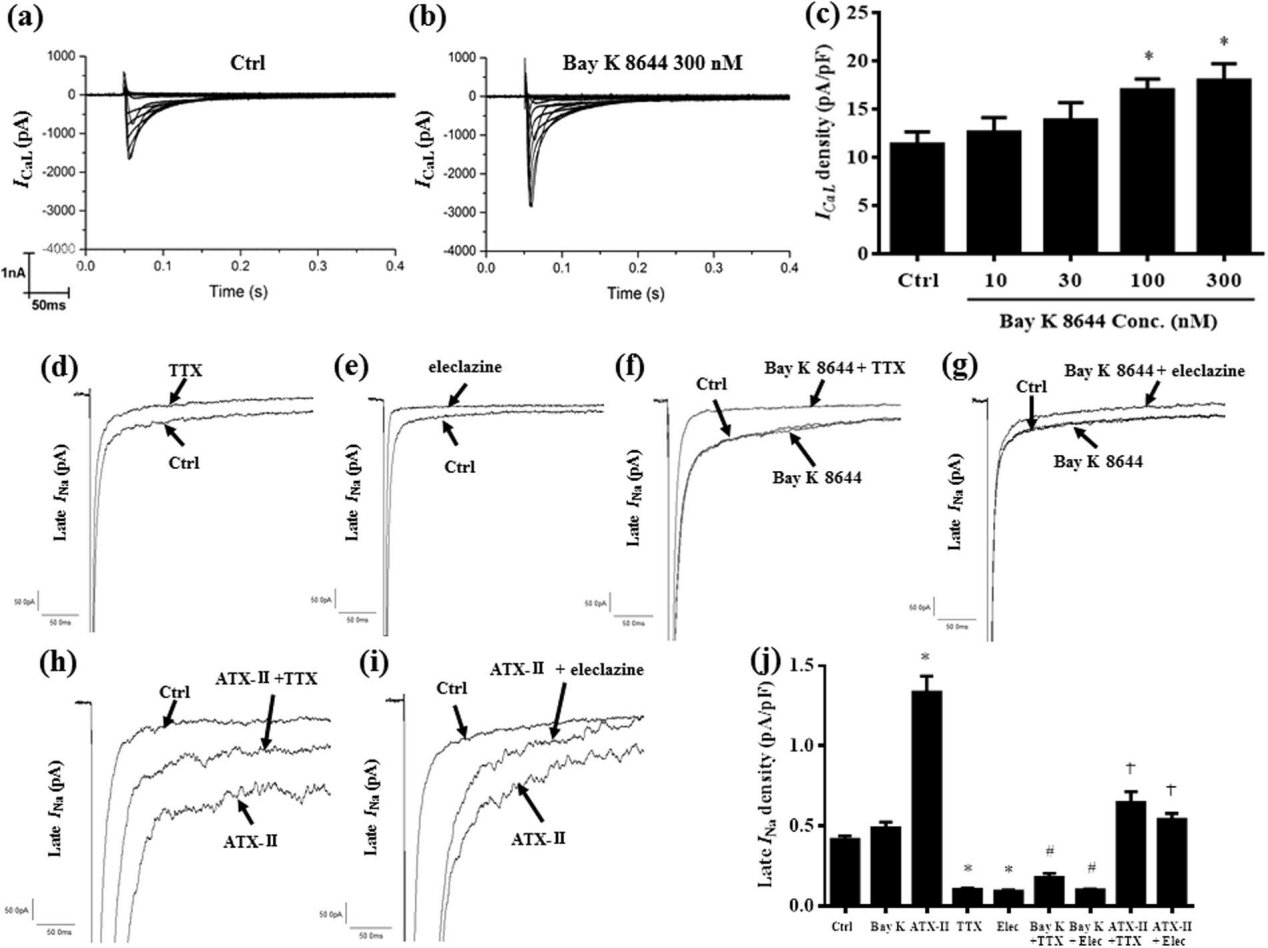
Bay K 8644 increased *I*_CaL_. TTX and eleclazine reduced both endogenous and enhanced late *I*_Na_. (**a**) and (**b**) Representative recordings of *I*_CaL_ in control (Ctrl, **a**) and in a cell treated with Bay K 8644 (300 nM, **b**). (**c**) Summarized data of various concentrations of Bay K 8644 to increase the density of *I*_CaL_ in rabbit ventricular cells. ^*^ *P* < 0.05 vs. control. (**d**) and (**e**) Representative recordings of late *I*_Na_ in a cell in Ctrl, and after treatment with either TTX (1 µM, **d**) or eleclazine (Elec, 10 µM, **e**). (**f**) and (**g**) Representative recordings of late *I*_Na_ in a cell in Ctrl, before (Bay K 8644 300 nM alone) and after treatment with either TTX (**f**) or Elec (**g**). (**h**) and (**i**) Representative recordings of late *I*_Na_ in a cell in Ctrl, before (ATX-II 3 nM) and after treatment with either TTX (1 µM, **h**) or Elec (10 µM, **i**) in the continued presence of ATX-II. (**j**) Summarized data of TTX and Elec on the density of late *I*_Na_ in Ctrl and in cells treated with either Bay K 8644 (300 nM) or ATX-II (3 nM). ^*^ *P* < 0.05 vs. Ctrl; ^#^ *P* < 0.05 vs. Bay K 8644; ^†^ *P* < 0.05 vs. ATX-II.

In isolated ventricular myocytes, Bay K 8644 (100 and 300 nM) significantly enhanced *I*_CaL_ in a concentration dependent manner (Fig. 6(a) and (b), n = 5). The current density of *I*_CaL_ was increased from 11.41 ± 1.29 to 17.06 ± 1.11 and 18.04 ± 1.72 pA/pF, respectively, by 100 and 300 nM Bay K 8644 (*P* < 0.05, n = 5). Late *I*_Na_ was significantly increased by ATX-II (3 nM) from 0.42 ± 0.02 to 1.34 ± 0.10 pA/pF (*P* < 0.05 vs. ctrl, n = 10), but not by 300 nM Bay K 8644 (0.49 ± 0.04 pA/pF, *P* > 0.05, n = 9). TTX and eleclazine significantly decreased late *I*_Na_ to 0.11 ± 0.01 and 0.09 ± 0.01 pA/pF, respectively (n = 5 for both groups, *P* < 0.05, vs. ctrl), to 0.65 ± 0.07 and 0.54 ± 0.04 pA/pF, respectively (n = 4 and 6, *P* < 0.05, vs. ATX-II), and to 0.18 ± 0.03 and 0.10 ± 0.01 pA/pF, respectively (n = 4 and 5, *P* < 0.05, vs. Bay K 8644).

### TTX reduced the phosphorylation of CaMK II-δ and the expression of Na_v_ 1.5 in hearts treated with Bay K 8644 and/or ATX-II

**Figure 7:**
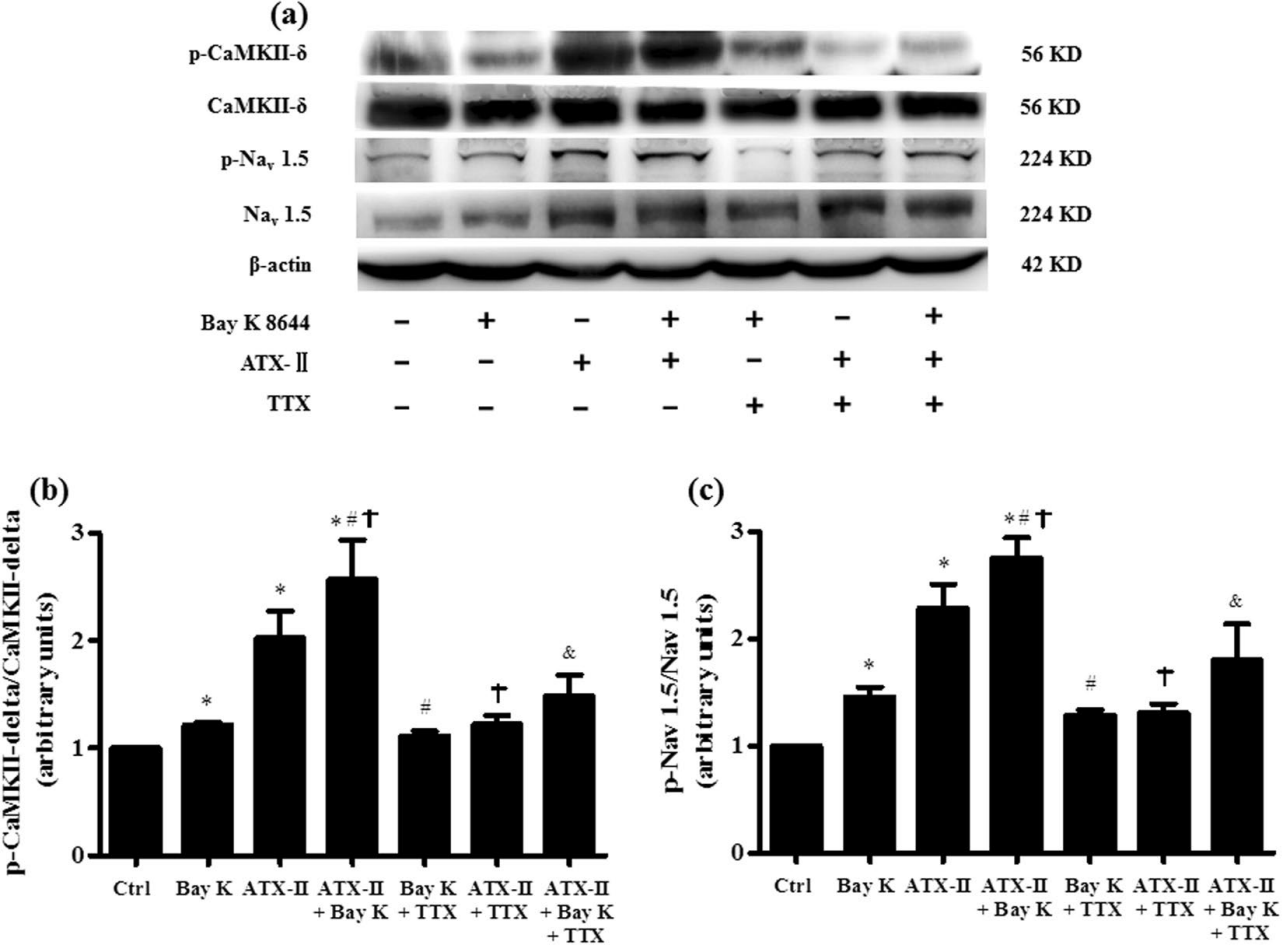
TTX (at concentration of 1 µM) reduced the phosphorylation of CaMK II-δ and Na_v_ 1.5 in hearts pretreated with Bay K 8644 (200 nM) in both absence and presence of ATX-II (3 nM). (**a**) Representative protein expression of p-CaMK II-δ, CaMK II-δ, p-Na_v_ 1.5 and Na_v_ 1.5 in rabbit left ventricular myocardium under conditions indicated. (**b**) and (**c**) The quantitative analysis of p-CaMK II-δ (**b**) and p-Na_v_ 1.5 (**c**) protein expression. The corresponding intensity was normalized to expression in Ctrl. ^*^ *P* < 0.05 vs. Ctrl; ^#^ *P* < 0.05 vs. Bay K 8644; ^†^ *P* < 0.05 vs. ATX-II; ^&^ *P* < 0.05 vs. ATX-II plus Bay K 8644.

The phosphorylation of CaMK II-δ and the expression of Na_v_ 1.5 in left ventricular myocardium was significantly enhanced in heart treated with either Bay K 8644 or ATX-II alone (n = 5, *P* < 0.05 compared with control, Fig. 7). The upregulation of p-CaMK II-δ and the expression of p-Na_v_ 1.5 were substantially greater in hearts treated with the combination of ATX-II and Bay K 8644 than in hearts treated with either Bay K 8644 or ATX-II alone. Inhibition of late *I*_Na_ by TTX attenuated the Bay K 8644-induced phosphorylation in CaMK II-δ and the expression of Na_v_ 1.5 (Fig. 7). Representative protein bands expression of p-CaMK II-δ and p-Na_v_ 1.5 in each group were displayed in Fig. 7(a).

## Discussion

The findings described in the present study suggest that endogenous and enhanced late *I*_Na_ play important roles in the electrical and mechanistic dysfunctions associated with increased [Ca^2+^]_i_ by activating CaMK-II and sodium channel activities. Bay K 8644 treatment, by increasing *I*_CaL_, induced prolongation of MAPD_90_, enhanced RUD, increased calcium transient and upregulation of phosphorylated CaMK II-δ and Nav 1.5, finally led to cardiac arrhythmias and contractile dysfunction. When late *I*_Na_ was amplified by ATX-II, Bay K 8644 caused greater increases in MAPD_90_ in a synergistic manner, higher incidence of ventricular arrhythmias, and greater phosphorylation of CaMK II-δ and Na_v_ 1.5. TTX and eleclazine, at concentration that inhibits late *I*_Na_, attenuated the pro-arrhythmic effects of Bay K 8644.

Cardiac Ca^2+^ homeostasis plays crucial roles in the maintenance of cardiac excitation and contraction function, and inhomeostasis of intracellular [Ca^2+^]_i_ is common in a number of pathological conditions. Ca^2+^ release from SR leads to arrhythmias in the setting of LQTs, CPVT and digoxin toxicity^21^. The increase in transmembrane inward *I*_CaL_ caused by mutations of L-type calcium channel or RYR2, is associated with LQTs 8^11^ and CPVT^22^ in clinic. Therefore, L-type calcium channel activator Bay K 8644 was used to mimic pathological conditions associated with increased [Ca^2+^]_i_ by amplifying *I*_CaL_ and stimulating Ca^2+^ influx, which could also reduce the repolarization reserve^23, 24^. Similar to our results, study from optical mapping data has indicated that abnormal Ca^2+^ handling caused arrhythmias in models of both repolarization impairment and bradycardia. The regional Ca^2+^ heterogeneities in Ca^2+^ handling leading to temporal instability of AP duration were considered one of the mechanisms for arrhythmias in the study^25^.

ase proarrhthmic in hearts when Endogenous late *I*_Na_ is small in amplitude (about 20–80 pA) in a normal heart and will be increased in many physiological (e.g., slow heart rate), pharmacological and pathological conditions^26^, and then the increase in endogenous late *I*_Na_ contributes to the arrhythmogenic activities in hearts with reduced repolarization reserve. Enhanced late *I*_Na_ caused by the mutation of SCN5A was initially reported to be contributed to arrhythmias in patients with LQTs 3. The “gain-of-function” of sodium channel prolonged ventricular repolarization and caused malignant arrhythmias. Heterogeneous distribution of late *I*_Na_ was also responsible for interregional differences in rate adaptation of repolarization, and is arrhythmogenic when rate dependence of repolarization was augmented^27^. Our previous study has revealed that the endogenous late *I*_Na_ is arrhythmogenic in hearts after use of *I*_Kr_ inhibitors, when repolarization reserve was reduced by decrease of outward current^19^. The role of endogenous late *I*_Na_ should be of concern in patients with calcium-related arrhythmias, especially in patients with bradycardia. RUD model of APD prolongation is an implicit property of several drugs that either increase inward current of repolarization or decrease outward current of repolarization. Our previous study has proved that endogenous late *I*_Na_ contributes to the increased RUD of APD and beat-to-beat variability of repolarization (BVR) induced by *I*_Kr_ inhibitors, and to bradycardia-related ventricular arrhythmias^14^. In the present study, the contribution of late *I*_Na_ was also found on enhanced RUD induced by Bay K 8644. The results suggesting that RUD augment of late *I*_Na_ is the basis of the increased RUD of MAPD_90_ in hearts with increased [Ca^2+^]_i_. The rate-dependent augment of late *I*_Na_ is the basis of the increased RUD of APD under repolarization reserve reduced condition, may explain the clinical phenomenon that LQTs 8 patients are often characterized by bradycardia, and late *I*_Na_ inhibitor is effective to treat LQTs 8^11^.

At the concentration of 3 µM or lower, TTX has been shown to be selective in inhibiting late *I*_Na_ with minimal inhibition on peak *I*_Na_ in rabbit cardiomyocytes^28^, which was confirmed in the present study that 1 µM TTX apparently decreased late *I*_Na_ in ventricular myocytes in both absence and presence of Bay K 8644 or ATX-II. In accordance with other’s report^20^, eleclazine exhibited a similar effect to TTX, inhibited late *I*_Na_, shortened the prolongation of MAPD, and attenuated ventricular arrhythmias. The shortening of MAPD and related antiarrhythmic activities induced by TTX and eleclazine in both absence and presence of either Bay K 8644 or ATX-II indicated that both endogenous and enhanced late *I*_Na_ were pro-arrhythmic in hearts when repolarization reserve was reduced by increase of Ca^2+^ current by Bay K 8644. Inhibition of endogenous late *I*_Na_ was sufficient in eliciting antiarrhythmic effects in hearts with increased [Ca^2+^]_i_. The underlying mechanism may be that endogenous late *I*_Na_ is, at least in part, responsible for the prolongation of MAP, which enhances the arrhythmogenic effects from an increase in [Ca^2+^]_i_. However, because the IC_50_ of TTX on Na_v_ 1.5 has been reported as 1 µM, peak *I*_Na_ may also be potentially affected and contribute to the decrease in Na_v_ 1.5 and the changes of a cardiac EP, if a concentration above 1 µM was used in the study.

Enhanced late *I*_Na_ synergistically increased the risk of arrhythmias in hearts with Bay K 8644 by activating CaMK II-δ and Na_v_1.5. ATX-II, at concentrations of 3 nM or lower, increases late *I*_Na_ in rabbit isolated myocytes, prolongs APD in the intact heart model^17, 29^. Therefore, 3 nM ATX-II was used to enhance late *I*_Na_ in this study. The results clearly showed that, in hearts treated with 3 nM ATX-II, Bay K 8644 (200 nM) caused greater prolongation of MAPD in association with an increased incidence of VT. This suggests a synergistic effect between increased [Ca^2+^]_i_ and enhanced late *I*_Na_ in potentiating the arrhythmogenic activities. The results in this study demonstrated that either Bay K 8644 or ATX-II increased the phosphorylation of CaMK II-δ and Na_v_1.5. TTX, at low concentration with inhibition of late *I*_Na_ but relatively minimal inhibition of peak *I*_Na_, reversed the activation of CaMK II-δ and Na_v_1.5. The increase in the phosphorylation of Na_v_ 1.5 and CaMKII induced by Bay K 8644 may cause enhancement of late *I*_Na_. However, the increase in the density of late *I*_Na_ after Bay K 8644 administration in this single cell study was small and insignificant. One of the possible reasons underlying the disparity between Na_v_ 1.5 and late *I*_Na_ may be attributed to the difference in exposure time of Bay K 8644. The exposure time of Bay K 8644 for late *I*_Na_ recording from single cells was 3 minutes which is shorter than the perfusion time of 30 minutes for Na_v_ 1.5 and CaMKII phosphorylation assays from whole heart experiments. Further study is needed to determine the time course for Bay K 8644 to cause a steady-state maximal effect on late *I*_Na_.

The proteins interaction between sodium channel activities and Ca^2+^ handling may contribute to the synergistic effect of ATX-II and Bay K 8644 on the prolongation of MAPD and ventricular arrhythmias. Changes of key Ca^2+^-handling proteins associated with arrhythmias involve activation of CaMK II, and phosphorylation of L-type Ca^2+^ channels, RyR2 (promoting Ca^2+^ release) and Ca^2+^-ATPase SERCA2a. CaMK II has been identified as an important activator of late *I*_Na_. In normal ventricular myocyte, Ca^2+^ overload at concentrations higher (0.6–1 µM) than that in this study could increase the late *I*_Na_ via activating CaMK II and PKC pathway^15^. Others showed Na_v_1.5-dependent persistent Na^+^ influx could activate CaMK II, which in turn phosphorylated Na_v_1.5, RyR2, and L-type Ca^2+^ channels, further promoting Na^+^ influx and Ca^2+^ release^30^. Thus, the interaction of late *I*_Na_ and calcium handling forms a positive feedback loop with progressive increases in intracellular calcium and sodium concentration, leading to spatial heterogeneity of calcium transients and trigger activities. Various studies have revealed that Ca^2+^-handling proteins are regulated by the balance between serine/threonine protein kinases and phosphatase activity, and several potential CaMK II phosphorylation sites have been identified. Using *Scn 5a* knock-in mouse models, sodium channel phosphorylated at Ser 571 was revealed to promote abnormal repolarization and intracellular Ca^2+^ handling, and increase susceptibility to arrhythmia^31^. Importantly, the phosphorylated antibody used in the present study could recognize Na_v_1.5 at serine 573 and threonine 17 loci, which is different from the results in mouse study^30^.

Besides late *I*_Na_, there are also other factors regulated calcium-related arrhythmias. Exchange protein directly activated by cAMP (Epac)2 has been recently recognized as key mediators that regulated arrhythmogenic SR Ca leak via CaMKII delta-dependent signaling pathway. A recent study demonstrated that the underlying mechanism of impaired Epac2 signaling-induced arrhythmias is due to ROS dependent activation of late *I*_Na_^32^. Therefore, selective inhibition of late *I*_Na_ provides a strategy to prevent arrhythmias under the condition of intracellular calcium overload.

The findings of the present study may explain why *I*_Na_ inhibitor is able to attenuate arrhythmias of LQTs 8^11^. Further research will be necessary in the mechanistic study of cardiac arrhythmias in models of myocardial ischemia/reperfusion and oxidative stress^2^, atrial fibrillation with intracellular Ca^2+^ handling abnormalities, because late *I*_Na_ was reported to be increased under these conditions^33^. Subsequent inhibition of endogenous and enhanced late *I*_Na_ shows feasible antiarrhythmic results. Novel late *I*_Na_ inhibitors, e.g., GS-967 or 6615^34^, may thus be useful in treating ventricular arrhythmias in patients with intracellular Ca^2+^ overload. However, as the conditions of Ca^2+^ overload and late *I*_Na_ amplification may come together, application of late *I*_Na_ inhibitors only might attenuate the part of repolarization abnormality from the contribution of late *I*_Na_ and therefore only reverse part of the complex conditions but may be sufficient to be antiarrhythmic in experimental models, as well as in clinic. Increased intracellular [Ca^2+^]_i_ in LQT 3 cardiomyocytes in which late *I*_Na_ was already enhanced has been reported with decreased amplitude of late *I*_Na_^35^, suggesting that the relationships between the [Ca^2+^]_i_ and late *I*_Na_ need to be further determined under pathological conditions. Indeed, data from an ischemia-reperfusion model (in supplement of this study) indicated a decreased infarct area in hearts treated with a late *I*_Na_ inhibitor ranolazine. Therefore, further study to evaluate the role of late *I*_Na_ in various disease conditions and the possible application of drugs that inhibit both late *I*_Na_ and Ca^2+^ handling is needed.

## Methods

### Female Rabbit Isolated Whole Heart Model

New Zealand White female rabbits, weighing 2.5–3.5 kg, were sedated using 6 mg/kg xylazine i.m. and 40 mg/kg ketamine i.m. and then anesthetized by a “cocktail” (ketamine 15 mg/kg + xylazine 4 mg/kg in 1.5 mL saline) i.v. via the marginal ear vein. The heart was excised and perfused in Langendorff mode at a rate of 20 mL/min with modified Krebs-Henseleit (K-H) solution warmed to 37 °C. The solution contains (in mM): 118 NaCl, 2.8 KCl, 1.2 KH_2_PO_4_, 2.5 CaCL_2_, 0.5 MgSO_4_, 2.0 sodium pyruvate, 5.5 glucose, and 25 NaHCO_3_. The atrioventricular (AV) nodal area was thermally ablated, and hearts were paced on the ventricular septum (close to His bundle)^14^. The use of animals in this study conformed to the “Guide for the Care and Use of Laboratory Animals” published by the United States National Institutes of Health (NIH publication No. 85–23, revised 2011), and the study protocols were approved by the Institutional Animal Care and Use Committee (IACUC) of Peking University First Hospital (J201325).

### MAP and ECGs recording

Epi- and endo- MAP from left ventricle were recorded. MAPs signals were displayed in real time and calculated by Cambridge Electronics Device Spike II software to determine the MAPD_90_. A pseudo 12-lead ECG was recorded using a circular Einthoven-Goldberger ECG electrode system (Harvard Apparatus, Inc., Holliston, MA, USA) connected to a Biopac Wilson ECG amplifier (Biopac, Goleta, CA).

### Determination of arrhythmic activities

MAP and ECGs were monitored continuously, as reported previously^19^. An EAD was defined as the depolarization during phase 2 and/or 3 of an MAP signal. An EVB was defined as a spontaneous beat occurring earlier than the next paced beat. VT was defined as a sequence of three or more consecutive spontaneous ventricular beats at a rate exceeding the pacing rate. A *torsade de pointes* ventricular tachycardia (TdP) was defined as a VT wherein the morphology of the QRS complexes in a 12-channel ECG record was continuously changing.

### Determination of concentration-response relationships of Bay K 8644 on electrophysiological parameters in the absence and presence of ATX-II

Hearts were paced at the pacing rate of 1.0 Hz. (S)-(-)-Bay K 8644 (Bay K 8644, 1546, Tocris Bioscience, USA) of 10, 30, 60, 100, 200 and 300 nM was administrated to the heart for 8 minutes or longer at each concentration until a steady state maximal effect was observed either in absence or presence of 0.1, 0.3, 0.6 and 1 µM TTX (1078, Tocris Bioscience, USA), and 10 µM eleclazine (synthesized at Gilead Sciences, USA). To test the effects of Bay K 8644 in the presence of ATX-II (Alomone labs, Cat# STA-700, USA), hearts were perfused with ATX-II for 20 min and then exposed to Bay K 8644 and TTX.

### Determination of RUD of APD induced by Bay K 8644

To measure the RUD of APD, hearts were paced at increasing CLs of 400, 500, 667, 1000, 1333 and 2000 ms for 2 to 4 minutes at each CL. After exposed to K-H buffer (control), hearts were treated with 200 nM Bay K 8644 in the absence and presence of 1 µM TTX and 10 µM eleclazine, allowing 10 minutes between increases in drug concentration to record a maximal steady-state effect. The stimulation protocol at CLs from 400 to 2000 ms and measurement of MAPD_90_ was repeated before and after each drug treatment.

### Measurement of myocytes contraction function and calcium transient

Rabbit left ventricular cardiomyocytes were isolated enzymatically, as described previously^36^. Then cardiomyocytes stored in regular Tyrode’s solution containing 1.8 mM CaCl_2_ were loaded with 0.5 μM fura-2-AM (Sigma) for 10 min at 25 °C. Myocyte contraction function and calcium transient were measured using IonWizard’s Transient system (IonOptix LLC, Milton, USA). The myocardial contraction function was evaluated by the magnitude of myocyte shortening/re-lengthening. The intracellular calcium transient was determined with the ratio of fluorescence intensity at 340 and 380 nm (FFI = 340/380 ratio). Intracellular Ca^2+^ fluorescence measurements were assessed using the electrically stimulated rise in intracellular Ca^2+^ (ΔFFI)^36^. The maximal fluorescence (F_max_) with 20 μmol/L ionomycin and the minimal fluorescence (F_min_) with 20 mmol/L EGTA were determined, respectively. The formula to calculate the intracellular Ca^2+^ concentration ([Ca^2+^]_i_) is as follows: [Ca^2+^]_i_ = Kd (R − R_min_)F_min_/(R_max_ − R)F_max_^37^.

### Recordings of *I*_CaL_ and late *I*_Na_ using whole cell patch-clamp techniques

Left ventricular cardiomyocytes were isolated from rabbit hearts. Whole cell *I*_CaL_ and late *I*_Na_ were obtained with a patch-clamp amplifier (EPC-10, Heka Electronic, Lambrecht, Pfalz, Germany), filtered at 1 kHz, and digitized at 10 kHz. All patch-clamp experiments were performed at a room temperature of 23 ± 1 °C. For *I*_CaL_ recording, the pipette solution contained (in mM): 80 CsCl, 60 CsOH, 0.65 CaCl_2_, 5 disodium creatine phosphate, 5 MgATP, 40 aspartic acid, 10 EGTA, and 5 HEPES (pH 7.3). The bath solution was the Tyrode’s solution. *I*_CaL_ was elicited by a 150-ms prepulse to −40 mV from a holding potential of −80 mV followed by a 300-ms depolarizing pulse from −40 mV to 0 mV (0.2 Hz). *I*_CaL_ was determined as the difference between peak inward current and the current remaining at the end of the 300-ms pulse^15^. For late *I*_Na_ recording, the pipette solution contained (in mM): 120 CsCl_2_, 1 CaCl_2_, 5 MgCl_2_, 5 Na_2_ATP, 10 TEA-Cl, 11 EGTA, and 10 HEPES (pH 7.3, adjusted with CsOH). The bath solution contained (in mM): 135 NaCl, 5.4 CsCl_2_, 1.8 CaCl_2_, 1 MgCl_2_, 0.3 BaCl_2_, 0.33 NaH_2_PO_4_, 10 glucose, 10 HEPES, and 0.001 nicardipine (pH 7.4). Late *I*_Na_ was recorded by a 300-ms depolarizing pulse to −20 mV from a holding potential of −90 mV^14^. The amplitude of late *I*_Na_ was measured at 200 ms after initiation of the depolarization step before (control, no drug), and 3 minutes after exposure to 3 nM ATX-II or 300 nM Bay K 8644 in absence and presence of TTX and eleclazine, respectively.

### Immunoprecipitation and Western Blotting Assay

To determine the levels of phosphorylation of CaMK II-δ and the expression of Na_v_ 1.5, 7 groups of heart tissue were properly used and analyzed after perfusion with either K-H solution in absence (control) or presence of Bay K 8644 (200 nM), ATX-II(3 nM), ATX-II (3 nM) + Bay K 8644 (200 nM), Bay K 8644 (200 nM) + TTX (1 μM), ATX-II (3 nM) + TTX (1 μM), ATX-II (3 nM) + Bay K 8644 (200 nM) + TTX (1 μM). Total protein of left ventricular myocardium was extracted, immunoprecipitation and Western blotting analysis were performed using a standard method. In brief, tissue lysates were first incubated with magnetic beads covalently conjugated with anti-phospho-Akt substrate (RXXS*/T*) rabbit mAb (Cell Signaling Technology, #8050) and anti-phospho-PKA substrate antibody (RRXS*/T*) Ab (Cell Signaling Technology, #9621) at 4 °C overnight, respectively. Sample buffer mixed with the washed beads was heated at 95 °C for 5 min and subjected to SDS-PAGE. Then the proteins were blotted with rabbit anti- CaMK II-δ Ab (Abcam, ab90445), and anti-Na_v_ 1.5 Ab (Abcam, ab56240), respectively. Blotted antibodies were visualized by HRP-conjugated mouse anti-rabbit IgG mAb (Cell Signaling Technology, #5127) and ECL detection system (Millipore). Densitometric analyses of blots were performed using the Quantity One image analyzer software (Bio-Rad, Richmond CA, USA).

### Statistical Analysis

Data of MAPD_90_ were plotted and analyzed with Prism version 5 (GraphPad Software, San Diego, CA) and all parameters were expressed as mean ± SEM. The significance of differences in measures before and after interventions in the same hearts was determined by repeated measures one-way or two-way ANOVA followed by the Student-Newman-Keuls test. *P* < 0.05 was defined as significant difference.

## Electronic supplementary material

Supplementary information

## References

[1] Perennec J, Willemin M, Pocholle P, Hatt PY, Crozatier B (1992). Cardiac ultrastructural abnormalities in Syrian hamsters with spontaneous cardiomyopathy or subjected to cardiac overloads. Basic research in cardiology.

[2] Prunier F (2008). Prevention of ventricular arrhythmias with sarcoplasmic reticulum Ca2+ ATPase pump overexpression in a porcine model of ischemia reperfusion. Circulation.

[3] Zhang C (2014). Microtubule-mediated defects in junctophilin-2 trafficking contribute to myocyte transverse-tubule remodeling and Ca2+ handling dysfunction in heart failure. Circulation.

[4] Yin G (2014). Arrhythmogenic calmodulin mutations disrupt intracellular cardiomyocyte Ca2+ regulation by distinct mechanisms. Journal of the American Heart Association.

[5] Zhang Y, Matthews GD, Lei M, Huang CL (2013). Abnormal Ca(2+) homeostasis, atrial arrhythmogenesis, and sinus node dysfunction in murine hearts modeling RyR2 modification. Front Physiol.

[6] Priori SG (2002). Clinical and molecular characterization of patients with catecholaminergic polymorphic ventricular tachycardia. Circulation.

[7] Splawski I (2004). Ca(V)1.2 calcium channel dysfunction causes a multisystem disorder including arrhythmia and autism. Cell.

[8] Venetucci L, Denegri M, Napolitano C, Priori SG (2012). Inherited calcium channelopathies in the pathophysiology of arrhythmias. Nature reviews. Cardiology.

[9] Sikkel MB, Hayward C, MacLeod KT, Harding SE, Lyon AR (2014). SERCA2a gene therapy in heart failure: an anti-arrhythmic positive inotrope. British journal of pharmacology.

[10] Coppini R (2013). Late sodium current inhibition reverses electromechanical dysfunction in human hypertrophic cardiomyopathy. Circulation.

[11] Gao Y (2013). Inhibition of late sodium current by mexiletine: a novel pharmotherapeutical approach in timothy syndrome. Circulation. Arrhythmia and electrophysiology.

[12] Wu L (2008). Role of late sodium current in modulating the proarrhythmic and antiarrhythmic effects of quinidine. Heart rhythm: the official journal of the Heart Rhythm Society.

[13] Wu L (2008). Augmentation of late sodium current unmasks the proarrhythmic effects of amiodarone. Cardiovasc Res.

[14] Wu L (2011). Late sodium current contributes to the reverse rate-dependent effect of IKr inhibition on ventricular repolarization. Circulation.

[15] Ma J (2012). Calmodulin kinase II and protein kinase C mediate the effect of increased intracellular calcium to augment late sodium current in rabbit ventricular myocytes. American journal of physiology. Cell physiology.

[16] Sanguinetti MC, Krafte DS, Kass RS (1986). Voltage-dependent modulation of Ca channel current in heart cells by Bay K8644. The Journal of general physiology.

[17] Wu L, Shryock JC, Song Y, Belardinelli L (2006). An increase in late sodium current potentiates the proarrhythmic activities of low-risk QT-prolonging drugs in female rabbit hearts. The Journal of pharmacology and experimental therapeutics.

[18] Lai D (2013). Stretch current-induced abnormal impulses in CaMKIIdelta knockout mouse ventricular myocytes. J Cardiovasc Electrophysiol.

[19] Wu L (2009). Reduction of repolarization reserve unmasks the proarrhythmic role of endogenous late Na(+) current in the heart. Am J Physiol Heart Circ Physiol.

[20] Zablocki JA (2016). Discovery of Dihydrobenzoxazepinone (GS-6615) Late Sodium Current Inhibitor (Late INai), a Phase II Agent with Demonstrated Preclinical Anti-Ischemic and Antiarrhythmic Properties. Journal of medicinal chemistry.

[21] Lei M, Wang X, Ke Y, Solaro RJ (2015). Regulation of Ca(2+) transient by PP2A in normal and failing heart. Front Physiol.

[22] Hsiao PY (2013). Gene mutations in cardiac arrhythmias: a review of recent evidence in ion channelopathies. The application of clinical genetics.

[23] Wolkowicz P (2014). Inhibitors of arachidonate-regulated calcium channel signaling suppress triggered activity induced by the late sodium current. Eur J Pharmacol.

[24] Satoh, H., Hayashi, H., Blatter, L. A. & Bers, D. M. BayK 8644 increases resting calcium spark frequency in ferret ventricular myocytes. *Heart and vessels* Suppl 12, 58–61 (1997). 9476545

[25] Nemec J, Kim JJ, Salama G (2016). The link between abnormal calcium handling and electrical instability in acquired long QT syndrome - Does calcium precipitate arrhythmic storms?. Prog Biophys Mol Biol.

[26] Zaza A, Belardinelli L, Shryock JC (2008). Pathophysiology and pharmacology of the cardiac “late sodium current”. Pharmacol Ther.

[27] Qi D (2015). Heterogeneous distribution of INa-L determines interregional differences in rate adaptation of repolarization. Heart rhythm: the official journal of the Heart Rhythm Society.

[28] Zhang S (2012). Sophocarpine attenuates the Na(+)-dependent Ca2(+) overload induced by Anemonia sulcata toxin-increased late sodium current in rabbit ventricular myocytes. Journal of cardiovascular pharmacology.

[29] Wu L (2004). Antiarrhythmic effects of ranolazine in a guinea pig *in vitro* model of long-QT syndrome. The Journal of pharmacology and experimental therapeutics.

[30] Yao L (2011). Nav1.5-dependent persistent Na+ influx activates CaMKII in rat ventricular myocytes and N1325S mice. American journal of physiology. Cell physiology.

[31] Glynn P (2015). Voltage-Gated Sodium Channel Phosphorylation at Ser571 Regulates Late Current, Arrhythmia, and Cardiac Function *In Vivo*. Circulation.

[32] Yang Z (2016). Epac2-Rap1 signaling regulates reactive oxygen species production and susceptibility to cardiac arrhythmias. Antioxidants & redox signaling.

[33] Nattel S, Harada M (2014). Atrial remodeling and atrial fibrillation: recent advances and translational perspectives. Journal of the American College of Cardiology.

[34] Antzelevitch C (2014). The role of late I Na in development of cardiac arrhythmias. Handbook of experimental pharmacology.

[35] Potet F, Beckermann TM, Kunic JD, George AL (2015). Intracellular calcium attenuates late current conducted by mutant human cardiac sodium channels. Circulation. Arrhythmia and electrophysiology.

[36] Qian C (2012). Resveratrol attenuates the Na(+)-dependent intracellular Ca(2+) overload by inhibiting H(2)O(2)-induced increase in late sodium current in ventricular myocytes. PloS one.

[37] Grynkiewicz G, Poenie M, Tsien RY (1985). A new generation of Ca2+ indicators with greatly improved fluorescence properties. The Journal of biological chemistry.

