# Supplementary material for: Inhibition of late sodium current suppresses calcium-related ventricular arrhythmias by reducing the phosphorylation of CaMK-II and sodium channel expressions
Source: Sci Rep. 2017 Apr 20;7:981. doi: 10.1038/s41598-017-01056-0 (PMC5430524; doi:10.1038/s41598-017-01056-0)
Supplement: Supplementary file 1 — Supplementary information [file 41598_2017_1056_MOESM1_ESM.pdf]

Supplementary information

Inhibition of late sodium current suppresses calcium-related ventricular arrhythmias by reducing the phosphorylation of CaMK-Ⅱ and sodium channel expressions

Xiao-Hong Wei[1,2], Shan-Dong Yu[1,2], Lu Ren[1,2], Si-Hui Huang[1,2], Qiao-Mei Yang[1,2], Ping Wang[1], Yan-Peng Chu[1,2], Wei Yang[1,2], Yan-Sheng Ding[1,2], Yong Huo[1,2,*], and Lin Wu[1,2,*]

[1]Department of Cardiology, Peking University First Hospital, Beijing, 100034, China

[2]Key Laboratory of Molecular Cardiovascular Sciences, Ministry of Education, Beijing, 100191, China.

[*]Corresponding authors: Lin Wu, wuepgroup@126.com; and Yong Huo, huoyong@263.net.cn.

Methods

Myocardial ischemia/reperfusion (I/R) model establishment and myocardial infarct size assessment in rats. To determine the effect of late sodium current inhibitors on a disease model (myocardial ischemia/reperfusion injury) associated with late sodium current, male Sprague-Dawley rats weighing 240-260 g were subjected to left anterior descending coronary artery (LADCA) occlusion by ligation for 30 min followed by reperfusion for 90 min. Animals were divided into three groups: Sham, I/R model, and ranolazine + I/R. Ranolazine (10 μM) was administrated by intravenous for 30 min before ischemia, and was continuously administrated till the end of reperfusion at a speed of 1 ml/h.

At the end of reperfusion, the LADCA was re-ligated, and 2 ml of 4% Evans blue (Sigma Aldrich, Missouri, USA) was injected into the right femoral vein via intravenous catheter to delineate the area at risk (AAR). Then the heart was excised at a 5 mm level above the apex cordis and sliced transversely into five sections (1 mm thick). The slices were incubated in 0.375% 2,3,5-triphenyltetrazolium chloride (TTC) (AMRESCO, Ohio, USA) at 37 ℃ for 15 min to identify the infarct size. AAR, infarct size and left ventricle (LV) were determined using Image-Pro Plus 5.0 software (Media Cybernetic, Maryland, USA).

Results

Eleclazine decreased the reverse use dependence (RUD) effect of Bay K 8644 on left ventricular epi-MAPD$_{90}$. As shown in Supplementary Fig. S1, eleclazine exhibited similar effect to TTX on Bay K 8644-induced RUD phenomenon of MAPD$_{90}$. Bay K 8644 (200 nM) prolonged epi-MAPD$_{90}$ at all stimulation rates (n=5, P<0.05), and the prolongation was greater at longer cycle length (CL)s (e.g., 75.8 ± 4.4 ms at a CL of 2000 ms) than at shorter CLs (19.6 ±

5.2 ms at a CL of 400 ms, P<0.05). In the continued presence of Bay K 8644 in same group of hearts, eleclazine significantly attenuated the RUD of epi-$MAPD_{90}$, especially at longer CLs (n=5, P<0.05).

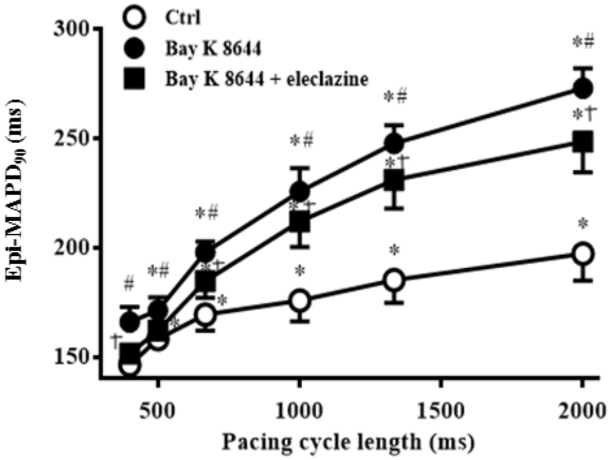

Supplementary Figure S1. Effects of eleclazine on the RUD of $MAPD_{90}$ caused by Bay K 8644. Values of $MAPD_{90}$ were measured in the absence (Ctrl) and presence of Bay K 8644 (200 nM), Bay K 8644 (200 nM) plus eleclazine (n=5). $^{*}P < 0.05$ vs. cycle length (CL) of 400 ms; $^{\#}P < 0.05$ vs. Ctrl at the same CL; †$P < 0.05$ vs. Bay K 8644 alone at the same CL.

Inhibition of late I_{Na} by ranolazine (10 μM) diminished the infarct size after myocardial I/R. As shown in Supplementary Fig. S2, ranolazine caused a reduced ratio of infarct size/AAR in hearts treated compared to that from I/R model (Supplementary Fig. S2, (a) and (c)). However, the ratios of AAR/LV were similar in all three groups, indicating similar tension and placement of the ligature across the three groups (Supplementary Fig. S2(b)).

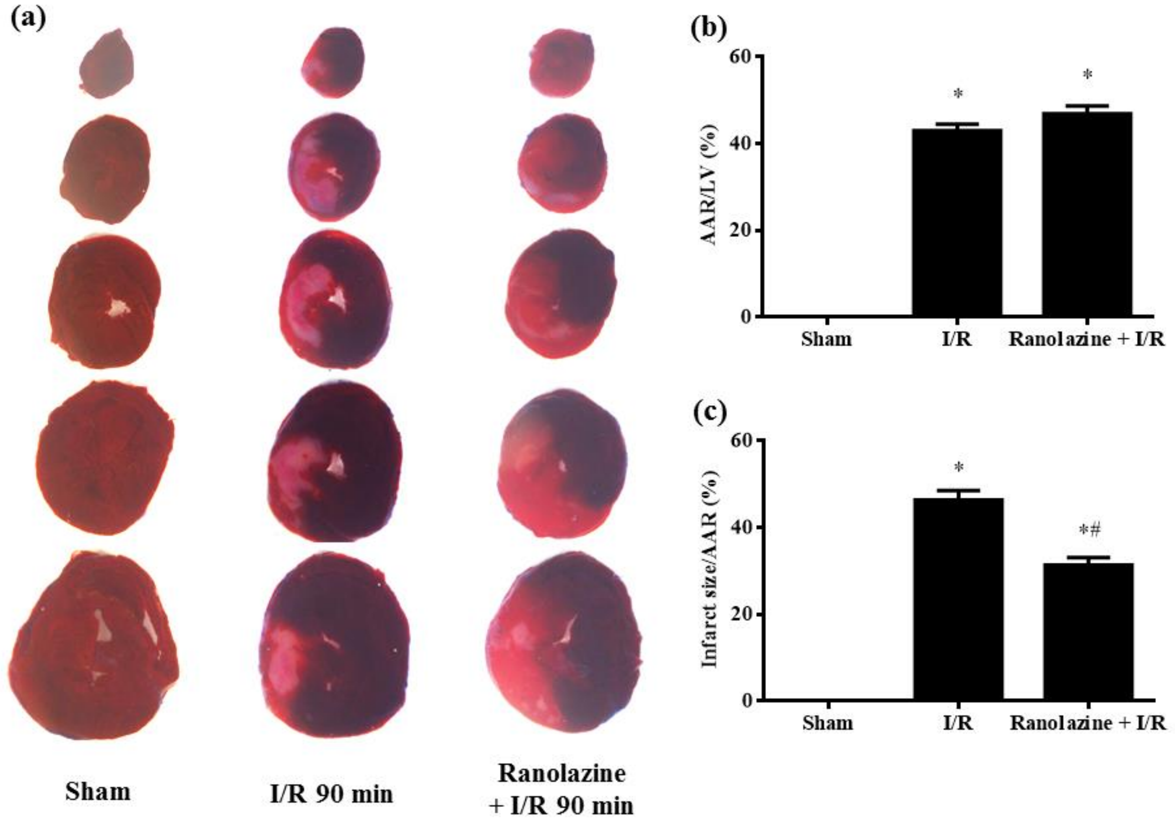

Supplementary Figure S2. Ranolazine (10 μM) reduced the infarct size in hearts with ischemia/reperfusion (I/R) injury. (a), representative images of Evans blue-TTX staining in Sham, I/R 90 min, and ranolazine plus I/R 90 min. (b) and (c), the statistic results of AAR/LV (b) and Infarct size/AAR (c) among the three groups. AAR: area at risk; LV: left ventricle. n=3, $^{*}P$ < 0.05 vs. Sham; $^{\#}P$ < 0.05 vs. I/R 90 min.
